# Subgingival Bacterial Microbiota Profile of People Living With HIV‐1 Under HAART: A Systematic Review

**DOI:** 10.1111/scd.70121

**Published:** 2025-11-24

**Authors:** Tatiana Pinheiro de Macedo, Cristiane Gonçalves, Lucio Souza Gonçalves, Priscila Pereira Pavan Vidal, Josué da Costa Lima‐Junior, Mario Vianna Vettore, Rodrigo Carvalho de Souza

**Affiliations:** ^1^ Postgraduate Program in Dentistry, Faculty of Dentistry, Estácio de Sá University‐IDOMED Rio de Janeiro Brazil; ^2^ Department of Dentistry and Oral Health Aarhus University Aarhus Denmark

**Keywords:** antiretroviral therapy, HIV‐1 infection, subgingival microbiota, systematic review

## Abstract

**Objective:**

This systematic review aimed to assess the available literature on the subgingival bacterial microbiota profiles of PLHIV undergoing HAART.

**Materials and Methods:**

This research was conducted in accordance with the PRISMA guidelines. Electronic searches were performed in the Cochrane Library, LILACS, PubMed, and Science Direct databases to identify primary studies evaluating the subgingival bacterial microbiota of individuals living with HIV‐1 undergoing HAART. The search was performed by two independent researchers and covered the period from January 2001 to July 2022. Studies published in English, Portuguese, and Spanish were included.

**Results:**

A total of 457 articles were initially retrieved. Of these, 9 met the eligibility criteria. The included studies revealed a high frequency of periodontal pathogens among individuals living with HIV‐1 on HAART, including *Treponema denticola* (44%, *n* = 4 studies) *and Porphyromonas gingivalis* (33%, *n* = 3 studies), as well as species not commonly found in patients with periodontal disease, such as *Enterococcus faecalis* (33%, *n* = 2 studies).

**Conclusions:**

According to the present findings, the most frequently identified subgingival bacterial species in patients living with HIV‐1 and receiving antiretroviral therapy were *T. denticola* and *P. gingivalis*, without establishing a consistent or unique microbiological profile.

## 1 Introduction

HIV‐1 infection remains one of the most significant public health problems worldwide. Although many advances have been made in the diagnosis, treatment, and prevention of the disease, a considerable number of deaths are still related to HIV‐1 infection [1]. In 2023, approximately 39 million people were living with HIV (PLHIV) worldwide [1]. Sub‐Saharan Africa continues to be the epicenter of the pandemic, accounting for nearly 60% of all new HIV‐1 infections globally.

The therapeutic regimen proposed for the management of HIV‐1 infection, known as highly active antiretroviral therapy (HAART), was introduced in 1995. Currently, there are 34 pharmaceutical formulations comprising 22 antiretroviral drugs available (Table 1) [2]. Since then, HIV‐1 infection has become a manageable chronic condition that has significantly reduced both mortality rates and opportunistic infections [1]. HAART mainly consists of the use of specific inhibitors of the viral replication cycle, which effectively reduce the viral load to undetectable levels for an extended period [3, 4]. Treatment with antiretroviral medication has shown constant evolution, and early initiation has been emphasized as a way to reduce mortality and disease progression. Additionally, HAART has proven to be an important tool in preventing HIV‐1 transmission [2].

**TABLE 1:** Antiretroviral drugs [2].

Item	Antiretroviral Drug (ARV)	Unit	Mechanism of action
1	Abacavir (ABC) 300 mg	Coated tablet	NRTIs
2	Abacavir (ABC) oral solution 20 mg/mL	Bottle	NRTIs
3	Atazanavir (ATV) 300 mg	Hard gelatin capsule	IP
4	Darunavir (DRV) 75 mg	Coated tablet	IP
5	Darunavir (DRV) 150 mg	Coated tablet	IP
6	Darunavir (DRV) 600 mg	Coated tablet	IP
7	Darunavir (DRV) 800 mg	Coated tablet	IP
8	Dolutegravir (DTG) 50 mg	Coated tablet	IP
9	Efavirenz (EFZ) 200 mg	Hard gelatin capsule	NNRTIs
10	Efavirenz (EFZ) 600 mg	Coated tablet	NNRTIs
11	Efavirenz (EFZ) oral solution 30 mg/mL	Bottle	NNRTIs
12	Enfuvirtide (T‐20) lyophilized powder for Injection 90 mg/mL	Ampoule‐bottle set	Fusion inhibitors
13	Etravirine (ETR) 100 mg	Coated tablet	NNRTIs
14	Etravirine (ETR) 200 mg	Coated tablet	NNRTIs
15	Lamivudine (3TC) 150 mg	Coated tablet	NRTIs
16	Lamivudine (3TC) oral solution 10 mg/mL	Bottle	NRTIs
17	Lopinavir + Ritonavir (LPV/r) 100 mg + 25 mg	Coated tablet	IP
18	Lopinavir + Ritonavir (LPV/r) 80 mg/mL + 20 mg/mL	Bottle	IP
19	Maraviroc (MVQ) 150 mg	Coated tablet	CCR5 inhibitors
20	Nevirapine (NVP) 200 mg	Simple pills	NNRTIs
21	Nevirapine (NVP) oral suspension 50 mg/5 mL–bottle with 100 mL	Bottle	NNRTIs
22	Raltegravir (RAL) 100 mg	Chewable tablet	Integrase inhibitors
23	Raltegravir (RAL) granule drug 100 mg	Sachet	Integrase inhibitors
24	Raltegravir (RAL) 400 mg	Coated tablet	Integrase inhibitors
25	Ritonavir (RTV) 100 mg	Coated tablet	IP
26	Ritonavir (RTV) 100 mg pó powder oral suspension	Set	IP
27	Tenofovir (TDF) 300 mg	Coated tablet	NRTIs
28	Tenofovir (TDF) 300 mg + Emtricitabine (FTC) 200 mg	Coated tablet	NRTIs
29	Tenofovir (TDF) 300 mg + Lamivudine (3TC) 300 mg (tablet comprising two in one)	Coated tablet	NRTIs
30	Tenofovir (TDF) 300 mg + Lamivudine (3TC) 300 mg + Efavirenz (EFZ) 600 mg (tablet comprising three in one)	Coated tablet	NRTIs + NNRTIs
31	Zidovudine (AZT) 100 mg	Hard gelatin capsule	NRTIs
32	Zidovudine (AZT) injectable solution 10 mg/mL	Ampoule‐bottle set	NRTIs
33	Zidovudine (AZT) oral solution 10 mg/mL–Frasco com 100 mL	Bottle	NRTIs

Abbreviations: IP, protease inhibitors; NNRTIs, non‐nucleoside reverse transcriptase inhibitors; NRTIs, nucleoside/nucleotide reverse transcriptase inhibitors

HAART is a treatment regimen typically comprising a combination of three or more antiretroviral drugs. The standard of care for most treatment‐naïve patients is a combination of two nucleoside reverse transcriptase inhibitors (NRTI) (typically tenofovir‐emtricitabine) plus one non‐nucleoside reverse transcriptase inhibitor (NNRTI) [5]. Currently, the first‐choice treatment is based on non‐boosted integrase inhibitors, which have a high rate of viral suppression and are well tolerated [4].

It is estimated that approximately one‐third of the entire global population living with HIV‐1 exhibits oral manifestations, which are more prevalent in countries with limited access to treatment [6]. Oral manifestations associated with HIV infection include oral candidiasis, herpes simplex virus infection, Kaposi's sarcoma, and periodontal disease, among others [6]. Of these, periodontal disease has been linked to HIV‐1 infection [7, 8, 9]. More specifically, necrotizing periodontal disease is associated with patients with HIV‐1 infection experiencing severe immunodeficiency, i.e., a low number of CD4 T lymphocytes, which are the primary target cells of the HIV virus [10].

Periodontitis is a chronic multifactorial inflammatory disease associated with dysbiotic biofilms and characterized by progressive destruction of the tooth‐supporting apparatus [11]. The host's inflammatory response to dental biofilm primarily involves neutrophils, monocytes/macrophages, and T and B lymphocytes. In this process, various inflammatory mediators such as cytokines and proteolytic enzymes are produced, contributing to tissue destruction [12].

The human microbiota and its relationship with various diseases have been extensively studied. The oral microbiota, in particular, has sparked significant interest among researchers who explore how dysbiosis can contribute to the development of oral diseases such as caries, periodontitis, and mucosal lesions, as well as its involvement in various systemic diseases [13]. Several studies have evaluated the periodontal microbiota in PLHIV and, more specifically, the subgingival microbiota of these patients under HAART [9, 14, 15]. It appears that HAART has a protective effect, resulting in low levels of pathogenic subgingival microbiota even in cases of immunosuppression [7].

Recently, two systematic reviews on the subgingival microbiota in PLHIV were published [9, 16]. The first systematic review investigated the prevalence of key bacterial pathogens in 22 primary studies comprising 965 HIV‐1 infected patients with periodontal disease [9]. A high prevalence of periodontal pathogens associated with the red and orange complexes, notably *Tannerella forsythia* (51%), *Fusobacterium nucleatum* (50%), and *Prevotella intermedia* (50%) was reported. Another review comprehensively analyzed how HIV‐1 infection and antiretroviral therapy influence the oral microbiome [16]. The authors hypothesized that periodontal disease possibly facilitates the establishment of an oral HIV‐1 reservoir, highlighting the need for further research on the gingiva's role in sustaining HIV‐1 persistence despite antiretroviral therapy. To date, no study has systematically reviewed the scientific literature on the subgingival bacteria profile in PLHIV undergoing HAART.

HAART has represented a milestone in the treatment of PLHIV. Since its introduction, antiretroviral medications have undergone significant progress. However, the impact of HAART on the subgingival microbiota of PLHIV remains poorly understood. The objective of this systematic review was to compile the available literature on this topic to answer the following research question: “What is the subgingival bacterial microbiota profile of people living with HIV under HAART?”.

## 2 Materials and Methods

The study was based on the items of the Preferred Reporting Items for Systematic Reviews and Meta‐Analyses (PRISMA) guideline [17]. The protocol of this systematic review was registered in the National Institute of Health Research Database (PROSPERO).

The studies selected for this systematic review met the following selection criteria: (1) Population: people living with HIV; (2) Exposure: antiretroviral therapy; (3) Comparison: not applicable; and (4) Outcome: bacteria more frequently detected in the subgingival sites of people living with HIV.

### 2.1 Search Strategy

The electronic searches were performed in four databases: Cochrane Library, LILACS, PubMed, and ScienceDirect. Additionally, the gray literature was searched using Google Scholar. A manual search was also carried out in the references of the selected papers. Studies in English, Portuguese, and Spanish that assessed the subgingival microbiota in PLHIV were selected. Two authors (T.P.M. and P.P.P.D.) conducted the search and selected original studies published between 2001 and 2022.

The following search terms were used in the electronic search and were combined using Boolean operators: (HIV/AIDS OR HIV infection OR HIV‐1 infection OR AIDS OR HIV OR HIV seropositive OR HIV positive OR HIV+) AND (chronic periodontitis OR periodontal OR periodontitis OR periodontal diseases) AND (microbiota OR microbiome OR microorganism OR bacteria OR periodontal pathogen OR oral microbiota OR oral microbiome OR biofilm OR bacterial community profiling OR microbial translocation).

### 2.2 Study Selection

Titles and abstracts of all retrieved papers were independently screened and assessed for inclusion by two authors (T.P.M. and P.P.P.D.). Any disagreements were resolved through discussion with a third author (RCS) to reach a consensus.

### 2.3 Eligibility Criteria

Original studies in humans assessing the subgingival microbiota of people living with HIV under HAART with periodontitis were included.

### 2.4 Data Collection Process

The following information was extracted for each paper: year of publication, author, country of origin, study design, sample size, molecular technique utilized, group, HAART type, and microbiological findings. The examiners demonstrated concordance with a kappa coefficient of 0.72.

### 2.5 Methodological Quality Assessment (Risk of Bias)

The methodological quality of all selected papers was synthesized using a detailed verification list: JBI critical appraisal checklist for quasi‐experimental studies [18], JBI critical appraisal checklist for analytical cross‐sectional studies [19], JBI critical appraisal checklist for case‐control studies [19], and JBI critical appraisal checklist for case series [20].

### 2.6 Data Synthesis

All extracted data were summarized and presented in descriptive tables.

## 3 Results

**FIGURE 1:**
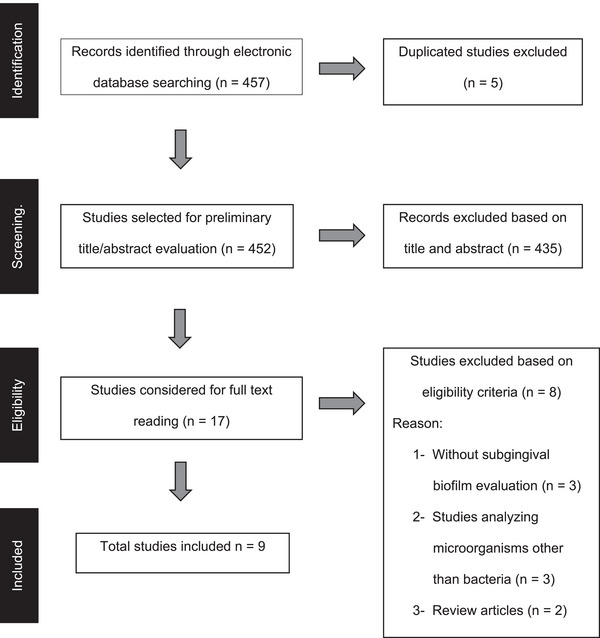
Flowchart of studies identification and selection.

A total of 457 papers were initially identified across four electronic databases: Pubmed (*n* = 432), 13 in ScienceDirect (*n* = 13), Cochrane (*n* = 9), and LILACS (*n* = 3). After removing duplicate studies (*n* = 5) and those not meeting the inclusion criteria based on title and abstract review (*n* = 435), 17 papers remained for full‐text evaluation. After reviewing the papers using the eligibility criteria, eight studies that did not assess bacteria, did not analyze subgingival biofilm, or were review papers were excluded. Finally, nine papers were included in this systematic review (Figure 1).

The study designs of the nine studies included were classified as follows: two quasi‐experimental, two case‐control studies, four cross‐sectional studies, and one case series (Table 2). Regarding HAART, only two studies did not detail the types of antiretrovirals used by the patients. The sample size of studies ranged from 11 to 172 participants.

**TABLE 2:** Selected papers.

Year	Authors	Country	Study design	Sample size	Molecular technique	Groups	HAART	Microbiological findings
2004	Gonçalves et al.	Brazil	Quasi‐experimental	64	DNA‐DNA	HIV with PD HIV no PD	Not specified	*Enterococcus faecalis* (28.5%), *Fusobacterium nucleatum* (37.5%) (moderate and severe immunosuppression)
2007	Gonçalves et al.	Brazil	Case‐control	172	DNA‐DNA	HIV with and no PD No HIV with and no PD	Lamivudine Zidovudine	*Acinetobacter baumannii* (40.0%), *Enterococcus faecalis* (77.5%), *Pseudomonas aeruginosa* (46.5%)
2008	Brito et al.	Venezuela	Cross‐sectional	32	PCR	HIV with PD No HIV with PD	NRTI + NNRTI IP	*Prevotella intermedia* (74.5%), *Aggregatibacter actinomycetemcomitans* (25.5%), *Porphyromonas gingivalis* (5.0%)
2009	Gonçalves et al.	Brazil	Case‐control	54	PCR	HIV with and no PD No HIV with and no PD	Lamivudine Zidovudine	*Enterococcus faecalis* (78.0%), *Pseudomonas aeruginosa* (45.0%)
2009	Vernon et al.	USA	Cross‐sectional	112	PCR	HIV with PD	2NUC + 1IP + NNRTI	*Porphyromonas gingivalis*, *Treponema denticola* *Tannerella forsythia*
2012	Ramos et al.	Brazil	Cross‐sectional	15	DNA‐DNA	HIV with PD	Stavudine	*Streptococcus intermedius* (76.7%), *Treponema denticola* (64.4%)
2016	Jordan et al.	Germany	Case series	11	PCR	HIV with PD	PI NNRTI	**PI**: *Actinomyces viscosus, Campylobacter rectus, Treponema denticola* **NNRTI**: *Fusobacterium nucleatum*, *Eikenella corrodens*
2019	Gliosca et al.	Argentina	Cross‐sectional	32	PCR	HIV with PD	Not specified	*Porphyromonas gingivalis*, *Treponema denticola* *Tannerella forsythia*
2022	Ramos Peña et al.	Brasil	Quasi‐experimental	32	PCR	HIV with PD No HIV with PD	NRTI	*Streptococcus alfa*, *Fusobacterium nucleatum*, *Veillonella parvula*, *Prevotella intermedia*

The microbiological findings of the included studies are reported in Table 2. Two molecular techniques were used to detect bacterial species in the subgingival biofilm. Checkerboard DNA‐DNA hybridization method was used in three studies [10, 21, 22], while six studies employed the polymerase chain reaction (PCR) method [14, 15, 23, 24, 25, 26].

The microbiological findings are described in Table 3. *Treponema denticola* was identified in four studies (44%, *n* = 4 studies) [10, 14, 15, 25], while *Enterococcus faecalis* [21, 22, 24], *F. nucleatum* [14, 21, 26], and *Porphyromonas gingivalis* [15, 23, 25] were each reported in three studies (33%). *Pseudomonas aeruginosa* [22, 24], *P. intermedia* [23, 26], and *T. forsythia* [15, 25] were detected in two studies each (22%). Other species were reported in only one study (11%).

**TABLE 3:** Overall frequency of bacterial species found in the selected studies.

Bacterial Species	Studies (*n* ^a^)	Sample size (*n* ^b^)	Frequency related *n* ^a^	References
*Treponema denticola*	4	170	44%	Vernon et al., 2009; Ramos et al., 2012; Jordan et al., 2016; and Gliosca et al., 2019
*Enterococcus faecalis*	3	290	33%	Gonçalves et al., 2004, 2007, and 2009
*Fusobacterium nucleatum*	3	107	33%	Gonçalves et al., 2004; Jordan et al., 2016; and Ramos Peña et al., 2022
*Porphyromonas gingivalis*	3	176	33%	Brito et al., 2008; Vernon et al., 2009; and Gliosca et al., 2019
*Pseudomonas aeruginosa*	2	226	22%	Gonçalves et al., 2007 and 2009
*Prevotella intermedia*	2	64	22%	Brito et al., 2008 and Ramos Peña et al., 2022
*Tannerella forsythia*	2	144	22%	Vernon et al., 2009 and Gliosca et al., 2019

^a^ Refers to the number of studies.

^b^ Refers to the sample size.

Risk of bias was assessed using JBI critical appraisal checklists for assessment of risk of bias for quasi‐experimental studies, cross‐sectional studies, case–control studies, and case series. Of the four cross‐sectional studies, three were at low risk of bias [15, 24, 26], while one study was at critical risk of bias [10]. Among those at low risk of bias, one was rated as having minor quality issues because confounding was not adequately addressed. The study at critical risk of bias had reduced quality mainly due to limitations in exposure measurement and inadequate control of confounding factors (Table 4). The two case‐control studies and the two quasi‐experimental studies were at low risk of bias (Tables 5 and 6). Lack of consecutive inclusion and completed inclusion of participants was detected in the case series study (Table 7).

**TABLE 4:** Methodological quality assessment of the cross‐sectional studies.

Evaluation criteria	Gliosca et al. 2019	Ramos et al. 2012	Vernon et al. 2009	Brito et al. 2008
1. The criteria for inclusion in the sample clearly defined	Yes	Yes	Yes	Yes
2. The study subjects and the setting described in detail	Yes	Yes	Yes	Yes
3. The exposure measured in a valid and reliable way	Yes	No	Yes	Yes
4. Objective, standard criteria used for measurement of the condition	Yes	Yes	Yes	Yes
5. Confounding factors identified	Yes	No	Yes	No
6. Strategies to deal with confounding factors	Yes	No	Yes	No
7. outcomes measured in a valid and reliable way	Yes	Yes	Yes	Yes
8. Appropriate statistical analysis used	Yes	Yes	Yes	Yes

**TABLE 5:** Methodological quality assessment of the case‐control studies.

Evaluation criteria	Gonçalves et al. 2009	Gonçalves et al. 2007
1. The groups are comparable, other than the presence of disease in cases or the absence of disease in controls	Yes	Yes
2. Cases and controls matched appropriately	N.A.	N.A.
3. The same criteria used for the identification of cases and controls	Yes	Yes
4. Exposure measured in a standard, valid, and reliable way	Yes	Yes
5. Exposure measured in the same way for cases and controls	Yes	Yes
6. Confounding factors identified	Yes	Yes
7. Strategies to deal with confounding factors stated	Yes	Yes
8. Outcomes assessed in a standard, valid, and reliable way for cases and controls	Yes	Yes
9. Exposure period of interest is long enough to be meaningful	Yes	Yes
10. Appropriate statistical analysis used	Yes	Yes

Abbreviation: N.A., Not applicable.

**TABLE 6:** Methodological quality assessment of quasi‐experimental studies.

Evaluation criteria	Penã et al. 2022	Gonçalves et al. 2004
1. It is clear in the study what the cause is and what the effect is	Yes	Yes
2. The participants included in any comparisons were similar	Yes	Yes
3. The participants included in any comparisons received similar treatment/care, other than the exposure or intervention of interest	Yes	Yes
4. There was a control group	Yes	Yes
5. There were multiple measurements of the outcome, both pre/post‐intervention/exposure	Yes	Yes
6. Follow‐up was complete, and if not, there were differences between groups in terms of their follow‐up adequately described and analyzed	Yes	Yes
7. The outcomes of participants included in any comparisons were measured in the same way	Yes	Yes
8. Outcomes were measured in a reliable way	Yes	Yes
9. Appropriate statistical analysis was used	Yes	Yes

**TABLE 7:** Methodological quality assessment of the case series studies.

Evaluation criteria	Jordan et al., 2016
1. There are clear criteria for inclusion in the case series.	Yes
2. The condition was measured in a standard, reliable way for all participants included in the case series.	Yes
3. Valid methods were used for the identification of the condition for all participants included in the case series.	Yes
4. The case series has consecutive inclusion of participants.	No
5. The case series has complete inclusion of participants.	No
6. There is clear reporting of the demographics of the participants in the study.	Yes
7. There is clear reporting of clinical information of the participants.	Yes
8. The outcomes or follow‐up results of cases are clearly reported.	Yes
9. There is clear reporting of the presenting site(s)/clinic(s) demographic information.	Yes
10. Statistical analysis is appropriate.	Yes

## 4 Discussion

This systematic review aimed to synthesize current evidence on the subgingival bacterial microbiota profile among people living with HIV (PLHIV) undergoing HAART. Nine studies in oral microbiology addressing the interaction between PLHIV and HAART were analyzed. The microbiological findings revealed that the following bacterial species were most frequently detected across the nine studies: *E. faecalis*, *F. nucleatum*, *Acinetobacter baumannii*, *P. aeruginosa*, *P. intermedia*, *Aggregatibacter actinomycetemcomitans*, *P. gingivalis*, *T. denticola*, *T. forsythia*, *Streptococcus intermedius*, *Actinomyces viscosus*, *Campylobacter rectus*, *Eikenella corrodens*, *Streptococcus alpha*, and *Veillonella parvula*. There was considerable variability in the frequency of subgingival species among the included studies. The complexity of this subgingival profile suggests that establishing a definitive periodontal microbiological pattern for PLHIV undergoing HAART is challenging, as no specific bacterial species were consistently detected across studies that characterized this population.

An important aspect to consider is that two different molecular techniques were employed in the microbiological analyses: three studies used checkerboard DNA–DNA hybridization [10, 21, 22], while six studies applied PCR [14, 15, 23, 24, 25, 26]. This methodological heterogeneity likely contributed to the variability in the subgingival profiles and represents a relevant limitation of the present review.

Two previous review papers were recently published on the same topic [9, 16]. A systematic review and meta‐analysis including 23 articles reported that more than 140 bacterial species were detected in PLHIV with periodontal disease [9]. The most frequently identified species were *T. forsythia* (51%), *F. nucleatum* (50%), *P. intermedia* (50%), *Peptostreptococcus micros* (44%), *C. rectus* (35%), and *Fusobacterium spp*. (35%) [9]. Only two studies included in that previous review [9] were also included in the present one [14, 23]. Similarly, *F. nucleatum*, *P. intermedia*, *A. actinomycetemcomitans*, *P. gingivalis*, *T. forsythia*, *C. rectus*, and *E. corrodens* were detected in both reviews.

In agreement, an integrative review comprising 29 studies on oral microbiota (bacteria, fungi, and viruses) in PLHIV [16] also identified overlapping species such as *F. nucleatum*, *P. gingivalis*, *T. denticola*, *C. rectus*, and *A. actinomycetemcomitans*. However, despite these similarities, numerous microbiological discrepancies remain, which may be attributed to methodological differences and variations in study populations. These findings are consistent with recent evidence showing that no single bacterial species or pattern consistently characterizes the oral microbiome of PLHIV under HAART [27].

The subgingival microbiota of PLHIV is influenced by systemic immune modulation and local ecological pressures. Immunosuppression due to reduced CD4+ T‐cell counts facilitates dysbiosis, favoring anaerobic and opportunistic pathogens [28]. In contrast, effective HAART improves immune competence, increases salivary IgA levels, and enhances neutrophil function, partially restoring microbial equilibrium [29]. However, immune reconstitution is often incomplete: chronic immune activation, altered cytokine profiles, and residual inflammation may persist even in virally suppressed individuals, perpetuating microbial imbalance and tissue breakdown [30].

The duration and composition of HAART regimens can differentially impact the oral microbiota. Prolonged therapy and higher CD4+ T‐cell counts have been associated with microbial profiles closer to those of HIV‐negative individuals, whereas shorter HAART duration or poor adherence maintains pathogenic dominance [27, 30]. Moreover, protease inhibitors and NNRTIs may indirectly affect the oral environment by altering saliva composition and metabolic activity, modifying bacterial colonization patterns [28, 29].

Clinically, this complex interplay between immune status, microbial ecology, and HAART underscores the need for individualized periodontal care in PLHIV. Patients with low CD4+ T‐cell counts or recent therapy initiation may require closer monitoring and preventive interventions to mitigate periodontal disease progression.

A notable limitation of the present review was the heterogeneity in microbiological detection methods. Studies employed checkerboard DNA–DNA hybridization and conventional PCR, each with different sensitivity and specificity. Such methodological variability likely contributed to the inconsistencies in bacterial detection and prevalence. Furthermore, most studies were cross‐sectional, limiting the ability to infer causality between immune parameters and microbial alterations. Differences in sampling protocols, disease definitions, and inconsistent reporting of HAART duration, CD4+ T‐cell count, and viral load further complicated data comparison [9, 16, 23, 24, 25, 26, 30].

Another relevant limitation concerns the nine studies included in that systematic review, which presented four different study designs: quasi‐experimental, case‐control, cross‐sectional, and case series. To address this heterogeneity among the included studies, a strategy was established following the Joanna Briggs Institute (JBI) Manual for Evidence Synthesis [18, 19, 20]. This framework allows the integration of diverse quantitative study types in systematic reviews when the research question cannot be addressed solely by randomized or homogeneous designs. Consequently, the heterogeneity among study designs was managed through qualitative synthesis and critical appraisal using JBI design‐specific tools, rather than through statistical pooling [18, 19, 20].

Future research should employ standardized, high‐throughput sequencing methods (e.g., 16S rRNA or metagenomics) to better characterize the microbial structure in PLHIV and its relationship with immune recovery [27, 30]. Longitudinal multicenter studies are needed to clarify how HAART duration, immune reconstitution, and host factors interact to modulate the oral microbiome. Integrating microbial, immunological, and clinical parameters could identify predictive biomarkers for disease susceptibility and treatment response, thereby guiding personalized care and informing public health strategies [28, 29, 30].

In summary, PLHIV under HAART harbor a diverse subgingival microbiota with no consistent bacterial profile, reflecting the complex interactions between systemic immunity, antiretroviral therapy, and local ecological factors. Understanding these interconnections is essential for improving periodontal care and promoting comprehensive oral health in this population.

## Author Contributions

**Tatiana Pinheiro de Macedo**: investigation, data curation. **Cristiane Gonçalves**: validation, supervision, conceptualization, methodology, writing—review and editing. **Lucio Souza Gonçalves**: validation, writing—original draft, writing—review and editing. **Priscila Pereira Pavan Vidal**: investigation, data curation. **Josué da Costa Lima‐Junior**: validation, writing—original draft, writing—review and editing. **Mario Vianna Vettore**: validation, writing—original draft, writing review, and editing. **Rodrigo Carvalho de Souza**: supervision, conceptualization, methodology, writing—review and editing. This systematic review is registered in PROSPERO under the number CRD42021265765 (http://www.crd.york.ac.uk/PROSPERO)

## Conflicts of Interest

All authors have no conflicts of interest to disclose.

## Data Availability

Data sharing not applicable to this article as no datasets were generated or analyzed during the current study.
